# Complement-Mediated Microglial Phagocytosis and Pathological Changes in the Development and Degeneration of the Visual System

**DOI:** 10.3389/fimmu.2020.566892

**Published:** 2020-09-24

**Authors:** Davis M. Borucki, Amer Toutonji, Christine Couch, Khalil Mallah, Baerbel Rohrer, Stephen Tomlinson

**Affiliations:** ^1^Medical Scientist Training Program, Medical University of South Carolina, Charleston, SC, United States; ^2^Department of Microbiology and Immunology, Medical University of South Carolina, Charleston, SC, United States; ^3^Department of Neurosciences, Medical University of South Carolina, Charleston, SC, United States; ^4^Department of Health Sciences and Research, College of Health Professions, Medical University of South Carolina, Charleston, SC, United States; ^5^Department of Ophthalmology, Medical University of South Carolina, Charleston, SC, United States; ^6^Ralph H. Johnson VA Medical Center, Charleston, SC, United States

**Keywords:** complement, synapse, phagocytosis, retina, lateral geniculate nucleus, multiple sclerosis, age-related macular degeneration, glaucoma

## Abstract

The focus of this review is the role of complement-mediated phagocytosis in retinal and neurological diseases affecting the visual system. Complement activation products opsonize synaptic material on neurons for phagocytic removal, which is a normal physiological process during development, but a pathological process in several neurodegenerative diseases and conditions. We discuss the role of complement in the refinement and elimination of synapses in the retina and lateral geniculate nucleus, both during development and in disease states. How complement and aberrant phagocytosis promotes injury to the visual system is discussed primarily in the context of multiple sclerosis, where it has been extensively studied, although the role of complement in visual dysfunction in other diseases such as stroke and traumatic brain injury is also highlighted. Retinal diseases are also covered, with a focus on glaucoma and age-related macular degeneration. Finally, we discuss the potential of complement inhibitory strategies to treat diseases affecting the visual system.

## Introduction

**Figure 1:**
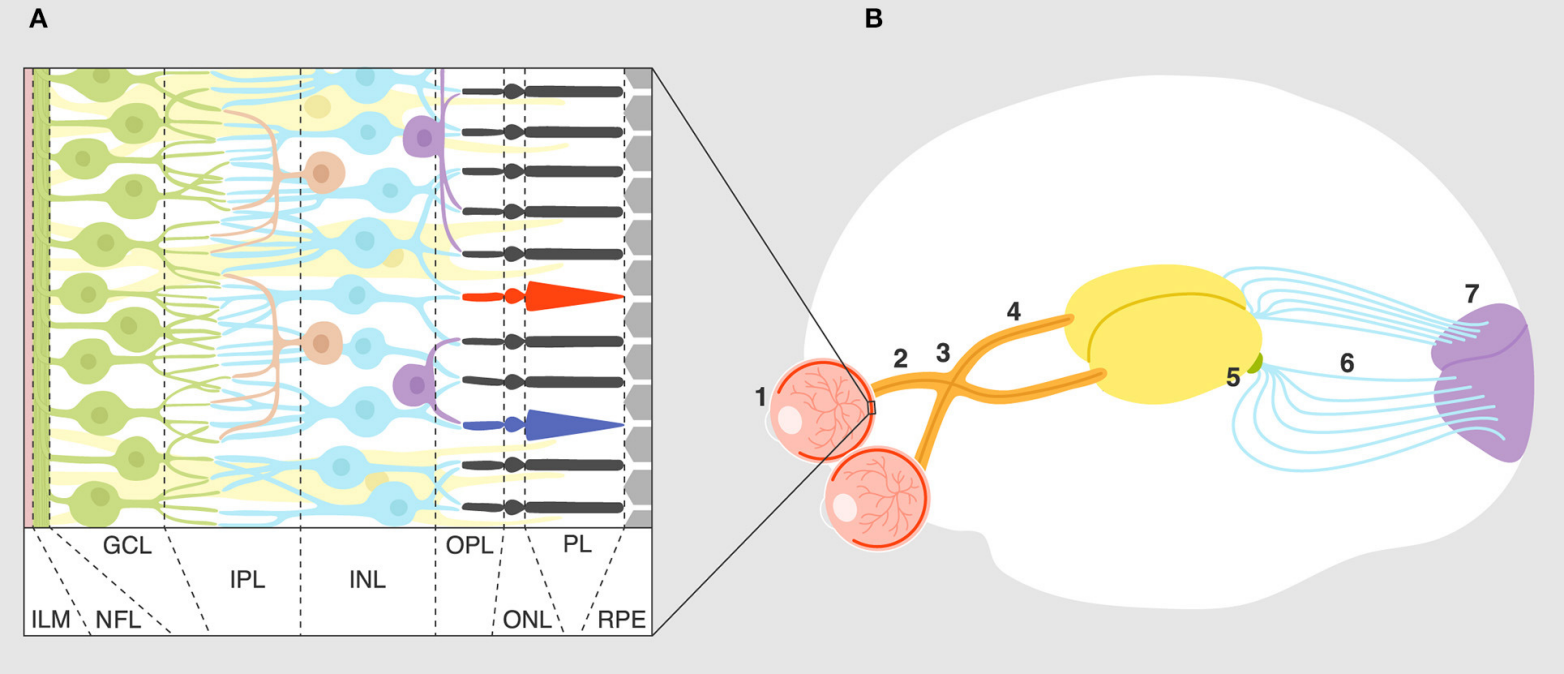
Overview of the visual system. **(A)** Layers of the retina: ILM, inner limiting membrane; NFL, nerve fiber layer; GCL, ganglion cell layer; IPL, inner plexiform layer; INL, inner nuclear layer; OPL, outer plexiform layer; ONL, outer nuclear layer; PL, photoreceptor layer; RPE, retinal pigment epithelium. Connections and cell types are explained in the text. **(B)** Connectivity of the visual system: eye (1), optic nerve (2), optic chiasm (3), optic tract (4), lateral geniculate nucleus (5), optic radiation (6), primary visual cortex (7).

The visual system is one of the best understood sensory/neural systems in terms of development and signal transduction and processing. The circuit itself is relatively simple (1); each part of a visual scene or light pattern is analyzed by photoreceptors that feed this information via bipolar cells to retinal ganglion cells (RGC). Interneurons (horizontal and amacrine cells) shape this response through excitation and inhibition. Signal is then passed via the optic nerve and optic tract by ganglion cell axons to the next synapse in the lateral geniculate nucleus (LGN). Neurons from the LGN terminate in the primary visual cortex for further visual processing of signal (Figure 1). As discussed later, the visual system in rodents develops and matures during the post-embryonic period for 2 weeks after birth (2). The fact that the rodent visual system develops postnatally makes it a useful experimental system for studying synaptic development and refinement.

Developmental studies of the visual system show an overproduction of RGC neurons in the retina and an abundance of synaptic connections in the developing LGN late in embryonic development (3). The complement system, and specifically the classical complement pathway, is necessary for this synaptic refinement and reduction of RGC numbers and for the division of the LGN into eye-specific subregions (4, 5). Studies of neurological injury, including mechanical, ischemic and autoimmune, show that the complement system plays a role in the propagation of damage after injury (6). The role of complement in injury to the visual system in these disorders is underexplored, but recent studies in experimental autoimmune encephalomyelitis (EAE), a model of multiple sclerosis (MS) with demyelination of the retinogeniculate system, synaptic changes and visual dysfunction (7), have indicated that complement-mediated phagocytosis of neural tissue is involved in injury (8). The retina, where the cell bodies of RGCs are located and where visual information is first encoded into a spike train of action potentials, is part of the central nervous system (CNS) affected physiologically and pathogenically by complement. During development, complement is involved in the synaptic pruning of RGCs and the sculpting of neural circuits (5). However, aberrant overactivation of the complement system in retinal disease can result in damage to RGCs with degradation and loss of synaptic connections on the retinal or thalamic side (4). The role of complement-mediated inflammation in retinal disease in general and anterograde degeneration of RGCs in particular is quite well explored, and several preclinical and clinical studies show that inhibiting specific complement pathways is protective (9–12).

## Complement and Phagocytosis

The complement system consists of about 50 soluble and membrane-bound proteins that include pattern recognition molecules (PRMs), enzymes, effector precursors, and regulatory proteins. Complement proteins act together to perform multiple physiological functions, such as detection and disposal of pathogens and cellular debris, while limiting off-target damage to normal tissue (13). The focus of this review is complement-dependent phagocytosis in the CNS, primarily in the visual system, but in this section, we also discuss the broader role of complement in pathologies of the CNS. Depending on the PRMs and enzymes involved, complement activation is classified into three canonical pathways: classical, lectin, and alternative. Whereas, the classical and the lectin pathway are initiated by PRMs C1q and MBL, respectively, the alternative pathway does not require a ligand-PRM interaction. Instead, the alternative pathway can spontaneously activate, but it also serves as an amplification loop for the classical and lectin pathways.

**Figure 2:**
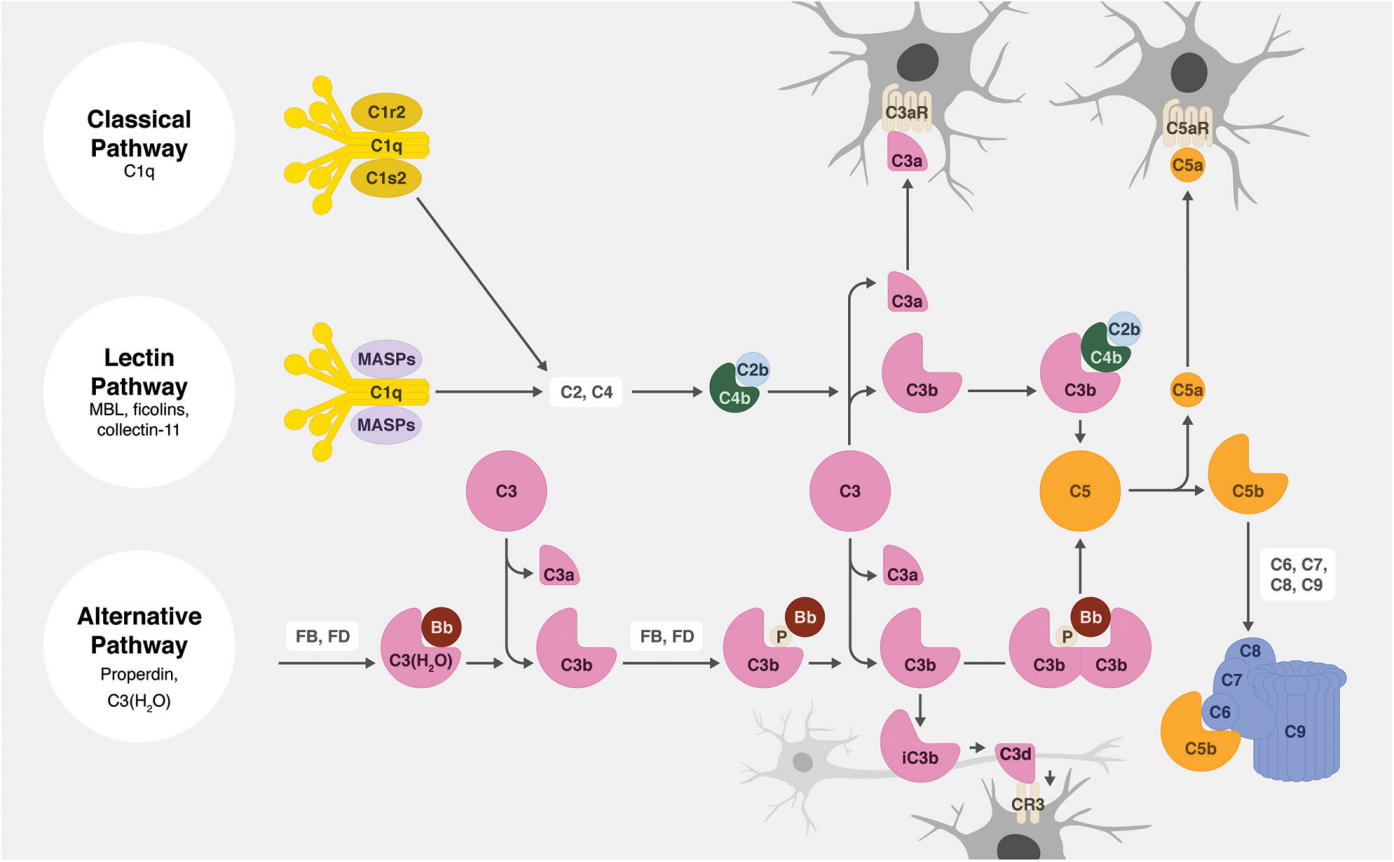
Overview of the complement system. There are three main complement activation pathways: the classical (top), lectin (middle), and alternative (bottom). All of these pathways lead to the cleavage of C3 and subsequently C5, leading to the opsonization of tissues (by C3b, iC3b, and C3d), the production of anaphylatoxins (C3a and C5a), and the assembly of the cytolytic membrane attack complex (C5b-9).

All activation pathways lead to the generation of biologic effector molecules, namely C3a, C3 opsonins, C5a, and C5b-9 (Figure 2). The C3a and C5a activation products are soluble anaphylatoxins that recruit and activate immune cells via interaction with their respective cellular receptors, C3aR and C5aR1. C5b-9, also known as the membrane attack complex (MAC), creates pores in the membrane that can lead to inflammatory signaling and/or cell lysis. The activation product C3b becomes covalently attached to activating surfaces and is the initial building block for the generation of enzymatic complexes that cleave C3 into C3a and C3b and cleave C5 into C5a and C5b. Furthermore, and of particular relevance to this review, surface-bound C3b is degraded to iC3b, C3d, and C3dg. These degradation products no longer act in the complement activation pathway, but they do function as cellular opsonins for complement receptors expressed on immune cells, including phagocytic cells. The effective clearance of synapses during circuit sculpting in development, and the removal of cellular debris and pathogens, necessary activities for normal development and for defense and recovery processes, is C3 opsonin driven.

The main phagocytic receptor for C3 opsonins is complement receptor 3 (CR3), which consists of the two integrin chains: CD11b (*itgax*) and CD18 (*itgb2*) (14). CR3 is mainly expressed on myeloid cells and is commonly used as a lineage marker for microglia, monocytes, and macrophages. The role of CR3 in microglial phagocytosis has been studied in the context of synaptic pruning (15), the clearance of myelin (16, 17) and amyloid β (Aβ) (18, 19), as well as the removal of several pathogens of the nervous system (20–22). In Alzheimer's disease (AD), for example, CD11b immunoprecipitation from homogenates of brain samples taken postmortem from the occipital lobe shows a significant presence of CD11b/C3b/Aβ complexes as compared to age-matched controls (23). In the amyloid precursor protein (APP) transgenic mouse model of AD, eliminating C3 resulted in increased levels of Aβ and loss of neurons in the hippocampus, indicating a beneficial role for complement-mediated phagocytosis in tissue homeostasis (24). This is in contrast with findings in more recent animal studies showing that CR3 deficiency improves microglial clearance of Aβ *in vivo* and *in vitro* by increasing enzymatic degradation (25). Another study, using the double transgenic human APP/PS1 (presenilin 1) AD model, shows that despite increased levels of cerebral Aβ upon elimination of C3, mice show better cognitive performance and fewer microglia around amyloid plaques along with reduced loss of neurons and synapses (26). Together, these seemingly contradictory findings could imply that C3 is important for the clearance of Aβ plaques, the effect of which depends on the presence of a mutant presenilin 1 (PS1) protein. In addition, these studies suggest that intracellular signaling via CR3 is proinflammatory and harmful in AD and that C3-triggered phagocytosis of Aβ is beneficial when mediated by receptors other than CR3. In this context, complement receptor 4 (CR4), typically a dendritic cell marker, has also been shown to be sharply increased with Aβ plaque load throughout all stages of AD. Furthermore, 23% of activated microglia are CR4+ in AD, suggesting a potentially important role for this receptor in Aβ clearance (27). CR3-mediated phagocytosis is also implicated in synaptic phagocytosis in the APP mouse model, suggesting that the same pathway leading to clearance of Aβ is also involved in synapse loss (28).

Complement-mediated phagocytosis is not restricted to C3 opsonins and CR3 and/or CR4 engagement. In addition to being the recognition molecule of the classical pathway, C1q can also bind directly to several membrane receptors including CR1, CD91, LAIR1, SCARF1, α2β1, cC1qR, and gC1qR (29–35), interactions that have been associated with complement-independent functions of C1q. There is evidence for C1q-mediated microglial phagocytosis in the clearance of apoptotic cells (36, 37). *In vivo* studies show that C1q deficiency rescues neurons in a frontotemporal dementia (FTD) model, and that C1q is involved in early synaptic pruning (38, 39). One study demonstrated that treatment of cultured rat microglia with extrinsic C1q triggers an increase in intracellular calcium that is associated with a shift toward proinflammatory activation (40). This study, together with another showing microglial expression of CD93 (41), indicate that direct C1q-mediated phagocytosis remains a viable pathway (42).

The complement anaphylatoxins (C3a and C5a) can also indirectly modulate phagocytosis. A recent presentation at the 2019 Society for Neuroscience conference reported that pretreatment of transformed mouse brain microglial cells (BV-2 cells) or primary mouse microglia with a C3aR agonist increased their motility and their ability to phagocytose beads and Aβ plaques. However, RNA sequencing attributed these findings to increased expression of genes involved in cell migration and proliferation, rather than phagocytosis (43). With regard to C5aR1, it has been shown that C5aR1 antagonism in a murine AD model improves histological and cognitive outcomes, and is associated with decreased inflammatory signaling and enhanced expression of degradation/clearance pathways (41). Detrimental, proinflammatory effects of C5aR1 signaling have also been shown in spinal cord injury (44). Importantly, anaphylatoxin effects are dynamic and context-dependent and should not be generalized. For example, an *in vitro* study showed that whereas short-lived exposure to C3a increases Aβ phagocytosis by primary microglia, chronic exposure attenuates Aβ phagocytosis, an effect that can be reversed by C3aR antagonists (45).

Finally, it is important to note that the mere presence of complement opsonins and receptors does not necessarily indicate ongoing phagocytic activity. Efficient phagocytosis also depends on recognition of ligands, intracellular signaling by multiple receptors, successful endosomal trafficking, lysosomal digestion and product recycling, and protection of surrounding cells from bystander cytotoxic effects (46). It has been shown that intracellular PI3K signaling triggered by Galectin-3 is crucial for activating CR3-mediated microglial phagocytosis of myelin (47, 48). Microglial CR3-mediated phagocytosis has also been shown to be dependent on DAP12, PKC, DAG, cAMP, MLCK, and Rho/Rock signaling pathways, all of which could be modulated pharmacologically or by other endogenous receptors (49–52). Moreover, knockout of Trem2, a receptor implicated in the phagocytosis of Aβ in AD, decreases expression of *C3* and *Cd11b* genes, in addition to other complement genes, indicating potential synergy between the two systems (53). Other microglial and astrocytic receptors also play a role in synaptic pruning and debris clearance independent of the complement system and have been the subject of other articles and reviews. These other receptors primarily involve TAM receptors (54), Trem2-APOE signaling (55), and the astrocytic MEGF10 and MerTK pathways (56, 57).

## Complement in the Development of the Visual System

Complement, and specifically the classical activation pathway, plays a key role in the postnatal development and refinement of synaptic connections in the visual system. Here we review the normal anatomy and prenatal and postnatal development within the visual system, specifically the retina-LGN-primary visual cortex axis, with an emphasis on the role of complement in the lateral geniculate nucleus.

RGC neurons have their cell bodies in the ganglion cell layer of the retina and project to, and synapse with, neurons in the LGN in retinally mapped (retinotopic) fashion. The RGC axons extend along the innermost layer of the retina (nerve fiber layer) to the optic disc, where they form the optic nerve and exit the posterior of the eye. Axons within the eye are not myelinated and become myelinated after exiting the eye and passing through the lamina cribrosa (58). Fibers from the nasal retina cross to the contralateral optic tract in the optic chiasm, such that objects on one side of the visual field will send all related activity from both eyes to the contralateral optic tract. In humans, 50–60% of fibers cross at the optic chiasm, while the percentage is closer to 90% in mice and rats (59). In humans, the majority of RGC axons project to the LGN, although some project or send additional projections to the hypothalamus, pretectum, or superior colliculus (60). In mice, <10% of RGC axons project to the LGN, with the vast majority terminating in the superior colliculus (61). Each mature RGC synapses with one or two neurons in the LGN (62). The LGN can be subdivided into the dorsal and ventral LGN, both of which receive input from the retina and the cortex. However, only neurons with their cell bodies in the dLGN send projections through the optic radiation to the occipital lobe and synapse in defined layers of the visual cortex. Neurons from the vLGN send projections to subcortical structures involved with motor tasks (63).

During prenatal development in the C57BL/6 mouse, retinal ganglion neurons send projections from the optic cup that grow anterogradely (toward the LGN). By embryonic day 14, fibers have crossed at the optic chiasm, and by embryonic day 16 have innervated the dLGN and superior colliculus. Parturition typically occurs at embryonic day 19 (64). By postnatal day 3, temporal fibers that do not cross at the optic chiasm enter the dLGN in a small region also innervated by crossed fibers. At this time, neurons in the LGN receive inputs from as many as 9-11 RGCs coming from both eyes (3). By postnatal day 8, competition between fibers leads to a decrease in synaptic density and, eventually, the establishment of two relatively well separated regions of the dLGN innervated by each of the two eyes (65). These regions are well segregated before natural eye opening at postnatal day 12–14, and by postnatal day 19, the number of RGCs converging on a LGN neuron is reduced to 1–3 from a single eye. This synaptic pruning process is caused by spontaneous retinal activity, which causes calcium influx that reinforces synaptic connections from some neurons and leads to the degradation of others (3). In a seminal 2007 publication, Stevens et al. demonstrated a definitive role for the classical complement pathway in C3-mediated postnatal synaptic pruning in the mouse LGN (4). C1q expression is induced in retinal ganglion cell neurons in response to TGF-beta (66) released from retinal astrocytes, and is localized to the inner plexiform layer in the retina and in the LGN early in postnatal development. C1q and C3 were found to colocalize with immature synapses during early postnatal development. Congenital deletion of the C1q A chain or of C3 leads to defects in eye-specific segregation within the LGN and increased innervation of LGN neurons by RGCs, suggesting that complement is involved in refining synaptic connections in this circuit. Congenital deficiency did not lead to any observable abnormalities in this region at postnatal day 5, suggesting that complement is not involved in axonal path finding or targeting in this region. Some pruning of RGC-LGN synapses, however, did occur in C1q or C3 knockout mice, suggesting that other opsonins and phagocytic receptors may play a role in synaptic pruning. Further studies showed that synaptic pruning in the postnatal LGN is CR3/C3-dependent, and that knockout of CR3 significantly attenuates synaptic phagocytosis and leads to defects in eye-specific segregation (15). Less active synapses are phagocytosed, but the exact mechanism by which C1q labels these synapses is not yet known, although C1q localization has been linked to apoptotic-like processes at synapses (39).

The above studies demonstrate that complement is necessary in normal postnatal brain development, which in addition to its mechanistic importance, indicates the potential benefits of targeting therapeutic complement inhibitors both in location and in time. For this purpose, we and others have investigated gene therapy approaches to correct glaucoma (10) or wet age-related macular degeneration (9) by overexpressing a complement inhibitor in the retina. In the brain, the application of complement inhibitors to reduce excessive synaptic pruning in the retinogeniculate circuit due to glaucoma would ideally need to be restricted to the LGN, as complement plays a positive role in synaptic phagocytosis and memory formation in the adult hippocampus (67). Complement-pathway gene expression by microglia is also higher in gray matter relative to white matter, which may be related to complement-mediated synaptic pruning under homeostatic conditions (68). From an experimental standpoint, the fact that complement has important roles in CNS development and homeostasis also has implications for the use of complement knockout mice in these studies, since synaptic architecture might be different between knockout and wild type mice, thus potentially confounding data interpretation. Lastly, the aforementioned study on the role of complement in RGC pruning in development and glaucoma also indicated that increased expression of complement proteins seen early in development and that are subsequently suppressed upon synapse maturation could again increase under certain pathologic/disease conditions.

## Complement in Demyelinating Diseases

The complement system plays a role in several demyelinating, and more broadly, neurodegenerative diseases of the CNS (69). Table 1 summarizes the diseases discussed here, and Table 2 summarizes the role of complement. Complement components are produced locally by neurons, astrocytes, and microglia, and can also access the CNS from the systemic circulation if there is blood-brain barrier (BBB) dysfunction (108). It is generally recognized that chronic inflammation is a characteristic of most neurodegenerative diseases (109). Symptoms of neurodegenerative disorders are caused in part by progressive loss of neuronal structures and functions until neuronal cell death leads to permanent disability. Here, we discuss and speculate on the role of complement in opsonizing neurons in the LGN, and myelin in the optic nerve, in demyelinating diseases of the CNS.

**Table 1:** Overview of the main aspects of visual system development and diseases discussed in this review.

**Disease**	**Main aspects**	**Pathology**	**References**
Development	During prenatal development, RGC axons send projections toward the LGN. By embryonic day 14, crossed fibers have innervated the LGN, and by postnatal day 3, uncrossed fibers have innervated the LGN. There are as many as 9–11 RGCs innervating each LGN neuron, but by postnatal day 19, the number of RGCs converging on a single LGN neuron has decreased to 1–3. Each eye also innervates distinct regions of the LGN.	n/a	(3, 64, 65)
Multiple Sclerosis	MS typically appears between 20 and 40 years of life, with a 2–3 times higher incidence in women. Neurological symptoms depend on the sites of inflammatory plaques and include sensory loss, visual dysfunction, muscle weakness, and cerebellar problems. The disease often follows a relapsing remitting course, with acute episodes of focal neurological deficits following by remission phases with full or partial recovery, but can also follow a progressive course in which neurological impairment is irreversible.	Inflammatory lesions cause demyelination and axonal loss in plaques that can occur throughout the CNS. Lesions are characterized by lymphocytic infiltration and gliosis. Demyelination is associated with deposition of Ig and complement	(70)
Neuromyelitis Optica	NMO onset peaks between 35 and 45 years of life, and has a 9–10 times greater incidence in women. Pathology is primarily restricted to the optic nerve and spinal cord. Relative to MS, NMO is more severe, with early and frequent relapses. Symptoms include vision and walking problems, with death sometimes occurring from respiratory failure.	There is vasculocentric deposition of immunoglobulin and complement activation products, thickened and hyalinized blood vessels, active lesions with perivascular inflammation and demyelination, chronic lesions with gliosis and degeneration	(71)
Myelin Oligodendrocyte Glycoprotein Antibody Disease	MOG-antibody disease has only recently been recognized as a distinct clinical entity. The disease can onset throughout life, with a median age of onset in the early 30s. It is slightly more common in women. Symptoms include optic neuritis, myelitis, or acute disseminated encephalomyelitis. The course of disease can be monophasic or relapsing. The outcomes tend to be better than in NMO.	Inflammatory lesions contain immunoglobulin and complement	(72)
Stroke	Stroke is a disruption of blood flow to a region of the brain that causes ischemia and acute infarction of tissue. The incidence rapidly increases with age. There is a greater age-adjusted incidence in men than women, although more women die of stroke each year due to their longevity. Clinical signs of stroke depend on the region affected but include focal limb weakness/paresis, facial paresis, speech disturbance, headache, gait problems, and visual problems.	Stroke is classified as ischemic infarction (87%), primary hemorrhage (10%), and subarachnoid hemorrhage (3%). In all cases, ischemia leads to neuronal injury and death with early cytotoxic edema. In hemorrhagic stroke, there are also cytotoxic blood products and possibly greater pressure-associated damage. Phagocytic cells infiltrate the lesion and lead to liquefaction of affected brain tissues. Several months after injury, astrocytes form a gliotic scar. There is often ongoing perilesional inflammation with complement deposition.	(73, 74)
Traumatic Brain Injury	Brain injury can occur after any blunt or penetrating blow to the head, including concussions and blast injury. The incidence is higher in older adolescents and older adults. Symptoms include physical complaints, headache, cognitive impairment, anxiety, irritability, and more.	Diffuse axonal injury occurs mainly at the gray-white matter junction and can lead to laminar necrosis. Cerebral edema and blood-brain barrier disruption can occur. Microglia and neutrophils infiltrate and phagocytose dead or dying tissue.	(75, 76)
Glaucoma	Glaucoma is the progressive loss of retinal ganglion cells and is the most common cause of irreversible blindness globally. It can be associated with normal or increased intraocular pressure. The incidence increases with age and is higher in women. The disease is often insidious, with pain only occurring with markedly increased intraocular presure and visual dysfunction only occurring late in disease.	Glaucoma is initiated by resistance to aqueous humor outflow from the eye (primary open-angle glaucoma) or obstruction of aqueous humor outflow (primary angle-closure glaucoma). However, increased pressure is not always seen in glaucoma, nor does increased pressure always cause glaucoma. There is impaired axonal transport in the optic nerve that leads to degeneration of RGCs. On exam, there is optic nerve cupping and retinal nerve fiber layer thinning.	(77–79)
Age-related Macular Degeneration	AMD affects at least 10 million Americans, and is a leading cause of blindness. The prevalence increases with age, with most disease occurring after age 55. The disease starts as dry AMD, with 10–15% of patients developing the wet form. The disease is progressive with gradual loss of central visual function. Geographic atrophy is an advanced form of dry AMD with more rapid vision loss.	Extracellular ocular deposits known as drusen, which accumulate normally with age, are elevated in AMD. Drusen accumulates between the retinal pigment epithelium and Bruch's membrane. There is progressive and irreversible loss of specific layers of the retina (photoreceptor layer, retinal pigment epithelium, and choriocapillaris). In wet AMD, choroidal neovascularization occurs and contributes to vascular leakiness, which further damages the retina.	(80)

*RGC, retinal ganglion cell; MS, multiple sclerosis; NMO, neuromyelitis optica; MOG, myelin oligodendrocyte glycoprotein; AMD, age-related macular degeneration*.

**Table 2:** Overview of the influence of complement in visual system development and in disease processes discussed in this review.

**Disease**	**Influence of complement**	**Model**	**References**
Development	C1q and C3 colocalize with presynapses in the early postnatal LGN	C1qA and C3 KO mice	(4)
	Complement-mediated synaptic pruning is CR3/C3-dependent	CD11b and C3 KO mice	(15)
	C1q colocalizes with apoptotic synapses	Proteomic analysis of wild type mice	(39)
Multiple Sclerosis	C3 opsonizes synapses in the LGN independently of C1q	Experimental Autoimmune Encephalomyelitis, Diphtheria toxin A demyelination	(8)
	Autoantibodies to ribbon synapses activate complement in the retina early in disease	Experimental Autoimmune Encephalomyelitis	(81)
	C1q colocalizes with synaptophysin, and C1q and C3 colocalize with microglial lysosomes in the hippocampus	Human postmortem brains	(82)
Neuromyelitis Optica	Antibodies to AQP4 activate complement	NMO patients' sera applied to mouse tissue	(83)
Myelin Oligodendrocyte Glycoprotein Antibody Disease	Antibodies to MOG activate complement	*In vitro* phagocytosis assay	(84)
Stroke	Natural IgM antibodies bind neoepitopes exposed after injury and activate complement on stressed neurons	Intraluminal filament Middle Cerebral Artery Occlusion	(85)
	Natural IgM antibodies bind neoepitopes exposed after injury and activate complement on stressed neurons	Microembolic Middle Cerebral Artery Occlusion, Intraluminal filament Middle Cerebral Artery occlusion	(86)
Traumatic Brain Injury	Complement activation products and local production of complement are observed in the penumbral area of resected brain within 82 h of TBI	Human resected brain	(87)
	Elevated complement proteins are detected in the plasma of TBI patients chronically (6 months) after TBI	Human plasma	(88)
	Chronic activation of the alternative pathway contributes to functional and cognitive deficits	Controlled Cortical Impact	(89)
Glaucoma	C1q and C3 upregulation occurs early in the development of glaucoma	DBA/2J mouse model of chronic glaucoma	(90)
	C3 and MAC are involved in RGC loss	Ocular hypertension mouse model via injection of a hyperosmolar solution into the left episcleral vein	(91)
	C1q and C3 bind directly to RGCs	Experimental glaucoma via microsphere injection into the anterior chamber	(92)
	The classical complement pathway is involved in early synaptic loss and dendritic atrophy	DBA/2J mouse model of chronic glaucoma, experimental glaucoma via injection of microbeads	(93)
	Complement activation and the MAC play a role in the apoptosis of RGCs	Ocular hypertension rat model via laser photocoagulation	(94)
Age-related Macular Degeneration	Polymorphisms in factor H, factor B, and C3 are associated with variable risk of AMD	Human genetic analysis	(95–97)
	The MAC is essential for the development of choroidal neovascularization	Laser-induced Choroidal neovascularization	(98)
	Activation of the alternative complement pathway is essential for the development of choroidal neovascularization	Laser-induced Choroidal neovascularization	(99)
	Upregulation of alternative complement pathway genes contributes to rod degeneration	Constant light exposure BALB/c mouse model	(100)
	C3d deposits on the RPE/Bruch's membrane and is required for rod and cone dysfunction, thinning of the retinal nuclear layers, and mitochondrial dysfunction	Chronic cigarette smoke exposure	(101)
	Complement activation products from both the classical and alternative pathways deposit on photoreceptor outer segments	Sodium-iodate induced blood-retina barrier disruption	(102)
	Drusen contains complement components C5 and MAC	Human postmortem retinal samples	(103)
	Complement anaphylatoxins produced in drusen contribute to neovascularization	Laser-induced choroidal neovascularization	(104)
	Phagocytic function in RPE cells declines in AMD and contributes to drusen accumulation and pathology	Human postmortem RPE cells *in vitro*	(105)
	Sub-lytic levels of the MAC on RPE cells contributes to inflammation and vascular leakiness	RPE cell line *in vitro*	(106)
	C3, factor B and MAC, but not C1q, deposit in the outer nuclear layer, outer segments, and RPE	Constant light exposure rat model	(107)

*RGC, retinal ganglion cell; LGN, lateral geniculate nucleus; KO, knock-out; MS, multiple sclerosis; CNS, central nervous system; NMO, neuromyelitis optica; MOG, myelin oligodendrocyte glycoprotein; TBI, traumatic brain injury; MAC, membrane attack complex; AMD, age-related macular degeneration; RPE, retinal pigment epithelium*.

Although the involvement of complement in MS has been known for decades (110), a pathological role for complement in the LGN in MS has been relatively unexplored until recently. Visual dysfunction has long been associated with MS, which commonly takes the form of optic neuritis, inflammation and demyelination of the optic nerve that can lead to permanent visual deficits and RGC loss (70). Recently, visual system degeneration in MS has been linked to polymorphisms in C3 and C1q genes, suggesting a role for complement (111). Werneburg et al. (8) recently demonstrated that in EAE models of MS, C3 colocalizes with synapses in the LGN, and that activated microglia consume synaptic material from both retinal and cortical afferents. C1q did not colocalize at synapses, leading the authors to conclude that synapse loss occurred via the alternative pathway. Notably, this process was inhibited by viral overexpression of CR2-Crry, a complement C3 inhibitor (Crry) that is targeted to sites of C3 opsonin deposition via its CR2 domain, in RGCs upon intravitreal delivery. The attenuation of vision loss in treated animals was attributed to preservation of synapses in the LGN, although a similar treatment protocol used in glaucoma suggests there would also be secretion of CR2-Crry in the retina (10), another site of complement-mediated pathology in EAE (112). It is not known if systemic administration of CR2-Crry will attenuate visual loss in EAE, but systemic administration does improve clinical scores in an EAE model (113). Jin et al. found that during the normal course of EAE there was axonal damage, but not significant neuronal/RGC death at the timepoint that Werneburg et al. observed synaptic phagocytosis, and that significant RGC death occurred later in the course of disease (112); it could thus be speculated that loss of synaptic connections in the LGN contributes to RGC death, triggering Wallerian or retrograde degeneration.

Multiple sclerosis is also accompanied by inflammation in the retina. While changes in vision in MS were previously thought to be due primarily to demyelination and inflammation in the optic nerve, recent studies suggest that complement activation on either side of the retinogeniculate pathway contributes to axonal loss. Dembla et al. (81) showed that autoantibodies directed against adhesion proteins in synapses in the retina are an early feature of EAE and possibly MS. These antibodies recruit complement to retinal synapses early in disease, and the presence of these antibodies causes visual dysfunction before noticeable demyelination or axonal loss in the optic nerve occurs, suggesting that demyelination may not be the initial cause of visual dysfunction in MS. In addition, the authors demonstrated that MAC colocalizes with synaptic markers, suggesting a role for the MAC in synapse loss. However, a role for C1q or phagocytosis was not investigated in this study, although both have been implicated in synaptic loss in other regions of the brain in MS (8, 82), as well as other retinal diseases (93).

Regional differences in microglia are also observed in MS (114). Within an active lesion, phagocytic cells are a mixture of microglia and macrophages, with the balance shifting toward the latter as the lesion progresses (115). These microglia display activated morphology and cellular markers. In an inactive lesion center, microglia express more anti-inflammatory markers. Within regions of normal-appearing white matter, there is debate as to whether nodules of microglia are activated or homeostatic (68, 115). These regional differences in microglial activation may relate to the different roles of microglia in the MS brain. The process of active demyelination in an active lesion contributes to a pro-inflammatory environment. Activated microglia in normal appearing white matter may represent early lesions or a response to Wallerian degeneration triggered by a distant lesion (116). Phagocytosis of myelin by microglia leads to a shift toward an anti-inflammatory pro-regenerative state (117), which is important for recovery and remyelination (118).

Complement and phagocytosis is involved in the clearance of myelin in MS and other demyelinating diseases. While this can occur in any region of the brain where there are active lesion, we focus here on optic neuritis. Optic neuritis is an autoimmune process characterized by transient inflammation and demyelination in the optic nerve, which causes visual dysfunction and eye pain upon movement. It is commonly associated with MS (119), although it is also a common feature of myelin oligodendrocyte glycoprotein (MOG)-antibody disease and neuromyelitis optica spectrum disorders (NMOSD) (120). MOG-antibody disease is an autoimmune demyelinating disorder characterized by an antibody against MOG, a component of the myelin sheath synthesized by oligodendrocytes. It frequently present with episodes of optic neuritis or transverse myelitis (72). NMOSD are a class of disorders that also present with optic neuritis or transverse myelitis (71), but are usually characterized by an antibody to aquaporin 4, which is expressed by astrocytes (83). In models of optic neuritis, astrocytes in the optic nerve express C3 (121), which also deposits on discarded myelin either directly or through complement-activating anti-MOG antibodies (84). In NMOSD models, although these antibodies do not directly bind myelin or oligodendrocytes, microglia/macrophages still phagocytose myelin debris (122). Unknown changes occur in the myelin of MS patients that contribute to enhanced phagocytosis (123). In these contexts, classically activated microglia are critical for myelin phagocytosis (114).

The complement system has been shown to have both deleterious and regenerative roles in the CNS. For example, although excess activation of the MAC leads to cell lysis, sub-lytic levels of the MAC have been shown to activate transcriptional programs favoring cell survival and resistance to apoptosis in oligodendrocytes (124) through increased calcium flux (125). Microglia/macrophage phagocytosis of myelin debris contributes to regeneration and remyelination by positively affecting oligodendrocyte precursor cell progression (126) and neurite outgrowth (127). Therefore, complement opsonization of myelin is essential for normal recovery from demyelinating episodes. The dual role of complement in clearance of debris on the one hand, and the clearance of functional synapses on the other, is an important consideration for any therapeutic strategy involving complement inhibition.

## Complement in Neurodegenerative Diseases

The role of complement in vision loss in other neurodegenerative disease states is less well investigated. After stroke, visual deficits are common and are estimated to occur in 65% of patients (128). Stroke can directly affect parts of the visual system, such as in posterior cerebral artery stroke (129), stroke affecting the optic radiations (130), or thalamic stroke (131). However, inflammatory changes and complement activation in areas remote to the primary event could also contribute to synaptic loss, either transsynaptically or via a shared vascular bed (for example, in the LGN). In this context, Alawieh et al. (86) showed that complement activation following stroke opsonized hippocampal synapses and resulted in a loss of synaptic density in perilesional areas, with associated cognitive decline that was ongoing for at least 30 days after stroke. It is possible that a similar complement-dependent inflammatory process contributes to visual decline after stroke.

In traumatic brain injury (TBI), up to 60% of patients experience some kind of visual dysfunction after injury, which can persist for at least a year after TBI (132). TBI patients exhibit a reduction in cell size and neuronal loss in nuclei of the thalamus following severe head injury, although the LGN itself has not been analyzed histologically (133). However, functional MRI of TBI patients shows decreased connectivity between the LGN and areas of the cortex that correlates with severity of injury (134). A case study of a TBI patient showed retrograde degeneration of RGCs 2 months after injury (135). These studies show that the thalamus, and specifically visual circuitry, is affected chronically following TBI. In murine models of TBI, axonal loss affecting the LGN is observed in both retinogeniculate (136) and corticothalamic pathways (137). Inflammation, specifically gliosis and morphological alterations in microglia, is observed in the LGN 1 week after injury (138). There is also ongoing BBB dysfunction in the thalamus up to 3 months after TBI (139). Some synaptic reorganization occurs in the LGN following TBI (140), suggesting that there are competing neurodegenerative and regenerative processes occurring. While direct injury to the retina (136) or optic nerve (138) following TBI can contribute to visual dysfunction, ongoing inflammation in the LGN and axonal loss is thought to contribute to vision loss and impair recovery.

The complement system plays an important role in a secondary neuroinflammatory injury phase after the initial mechanical insult to the brain. Complement activation products, including the MAC and C3 degradation products, have been detected in postmortem brain tissue of TBI patients in close proximity to neurons (87). Elevated levels of complement proteins have been detected in the plasma of TBI patients for up to 6 months after injury, suggesting a continuous role for complement in chronic inflammation (88). In a murine model of TBI, specific inhibition of MAC reduced acute deficits, but only inhibition of complement upstream at the C3 activation step provided chronic protection (89). In addition, this study also highlighted that the alternative complement pathway plays a particularly important role as an amplification loop in chronic neuroinflammation post-TBI. However, whether there is opsonization of neurons in visual circuits that are related to delayed visual deficits after TBI has not been investigated; nevertheless, given the pathological opsonization of synapses in various disease models, it is possible that aberrant complement activation in the LGN contributes to visual dysfunction following TBI.

In summary, the role of complement in neuroinflammation is well established, but its role in visual deficits resulting from neuropathological disease states is much less well characterized. Studies modeled around analyses within the LGN have established complement-mediated phagocytosis as an important mechanism involved in CNS development, and more recently, to be a mechanism involved with visual decline in models of MS. That the alternative pathway is implicated in synaptic phagocytosis in the LGN in MS comes as somewhat of a surprise, since MS is associated with myelin-recognizing autoantibodies capable of activating complement (141), and that the classical pathway mediates hippocampal synaptic phagocytosis in MS (82). However, the classical complement pathway is involved in synaptic loss in other diseases such as AD, as well as in other regions of the brain in MS (28, 82), and both classical and lectin pathways are implicated in stroke (85, 86). There remains a paucity of data on the role of complement in opsonophagocytosis within the LGN and visual circuitry after more generalized CNS injury, even though complement-mediated synaptic phagocytosis is known to occur in other areas of the brain in injury models with accompanying visual dysfunction.

## Complement in Diseases of the Retina

The role of complement in development and diseases of the retina is somewhat better characterized. During normal embryonic development, the classical complement pathway contributes to microglia-mediated RGC elimination (5), which reflect findings within the LGN (see above), and indicates that complement and microglia contribute to neuronal development and refinement in multiple regions relevant to the visual system. Of note, complement factor H-like protein 1, a truncated form of factor H, is expressed in the retinal pigment epithelium and spreads throughout the extracellular matrix in this layer of the retina (142). This protein is an alternative pathway inhibitor that functions at the C3 activation step and will thus inhibit C3 opsonization of tissues in the retina. The complement system has been implicated in several diseases of the retina, and for representation we focus here on only glaucoma and age-related macular degeneration, in which complement has a firmly established role and in which complement-based therapeutics have shown efficacy.

Glaucoma is a family of eye diseases characterized by damage to the optic nerve and vision loss (77). Complement component C1q is known to be upregulated in the retina during glaucoma (4), and upregulation of C1q and C3 expression is an early event in the development of glaucoma in mice (90, 91). C1q and C3 bind to RGCs in glaucomatous eyes in the absence of IgG: however, the target(s) for complement protein binding expressed by stressed RGCs is unknown (92). In experimental glaucoma, complement activation via the classical pathway leads to synaptic pruning in the retina by resident glial cells (93) as well as MAC-induced apoptosis of RGCs (94). Thus, the complement system appears to represent a viable therapeutic target in glaucoma. AAV-mediated expression of soluble C3d-targeted CR2-Crry by retinal ganglion cells reduces neuronal loss in a glaucoma model (10), in part by inhibiting phagocytosis of opsonized RGC synapses. Inhibition of C5 via intravitreal injection of an anti-C5 monoclonal antibody also reduced neuronal loss (11), indicating that both C3 activation products and terminal pathway products (C5a and/or MAC) may contribute to RGC loss.

Age-related macular degeneration (AMD) is another disease that can cause irreversible vision loss. It can be broadly divided into two forms: dry AMD and wet AMD. Dry AMD is characterized by progressive damage to the macula resulting from damage to the retinal pigment epithelium and choriocapillaris complex followed by photoreceptor cell loss, which results in progressive loss of central vision. Wet AMD, which sometimes follows dry AMD but can occur on its own, is characterized by the addition of VEGF-stimulated neovascularization that can grow under the retina. Drusen, an extracellular aggregate of protein and lipid and laminar deposits in Bruch's membrane, are often associated with both forms of AMD (80). Polymorphisms in multiple complement proteins including factor H (95), factor B (96) and C3 (97) are associated with increased or decreased risk of AMD, which provide strong support for the role of complement in AMD.

The alternative complement pathway and MAC have been shown to contribute to ocular injury in multiple AMD mouse models. These include the wet AMD model of laser-induced choroidal neovascularization (CNV) (98, 99), although the significance of complement is somewhat debated (143), and the dry AMD models of light damage (100), smoke exposure (101, 144) and sodium iodate-induced blood-retina barrier disruption (102). Drusen in the human retina contain complement components (103), and anaphylatoxins produced from complement activation contribute to the development of neovascularization in the mouse CNV model (104). A decline in phagocytic function of retinal pigment epithelium cells contributes to drusen and lipofuscin accumulation and AMD pathology (105). Also, sub-lytic levels of the MAC on the retinal pigment epithelium causes calcium flux that contributes to epithelial dysfunction and leakiness (106). Synaptic changes occur in the retina in AMD, specifically the retraction of photoreceptor synapses from the outer plexiform layer to the outer nuclear layer and the outgrowth of bipolar cell dendrites to this new location (145). While the mechanism of synaptic change is unclear, it has been demonstrated that C3 deposits in the outer nuclear layer in a murine model (107). It is not clear if C3-mediated phagocytosis is involved in these synaptic changes in AMD, especially since C3 does not appear to deposit in the outer plexiform layer, which is the location of the photoreceptor-to-bipolar cell synapses. In clinical trials, systemic administration of an anti-C5 monoclonal antibody did not slow the rate of progression of AMD (146), whereas intravitreal administration of C3 inhibitor pegcetacoplan did (12). However, the intravitreal administration of an alternative pathway inhibitor (anti-factor D blocking antibody) did not reach its clinical endpoint in two phase 3 trials (147). The different routes of administration notwithstanding, clinical data thus far suggest that C3 activation products, possibly C3 opsonins, may have a pathogenic role in AMD and that MAC formation contributes to macular injury.

Complement is implicated in many other diseases of the retina. To mention just two, upregulation of C3 and factor H in proliferative diabetic retinopathy suggests a role for the alternative complement pathway in this disease, and colocalization of C3 and microglia suggests the possibility of C3-mediated phagocytosis occurring in the retina (148). Classical complement pathway genes are upregulated in models of retinopathy of prematurity (149) and in the vitreous humor of human babies with this disorder (150). To our knowledge, the role of complement opsonizing retinal tissue in this condition has not been explored. Diabetic retinopathy and retinopathy of prematurity are both proliferative retinal diseases characterized by increased angiogenesis and vascular leakiness and inflammation, not unlike wet AMD.

## Complement Inhibitors in Retinal and Neurologic Disease

The involvement of complement in neurodegeneration in retinal diseases, and the importance of visual function with regards to quality of life, make development of complement therapeutics an important area of study in the field of complement. A detailed understanding of complement-dependent mechanisms involved in injury to the visual circuitry, including understanding the importance of C3 opsonins and receptor recognition, will be critical for establishing an optimal complement inhibitory strategy to treat retinal and neurodegenerative disease states. A summary of the studies referenced is included in Table 3.

**Table 3:** The complement targets of the therapeutic approaches discussed in this review.

**Disease**	**Target**	**Experimental model(s)**	**Description**	**References**
Multiple Sclerosis	C3	Experimental Autoimmune Encephalomyelitis, Transfer of encephalitogenic T cells	Systemic administration of CR2-Crry or CR2-fH improved clinical score and delayed onset of disease	(113)
	C3	Experimental Autoimmune Encephalomyelitis, Diphtheria toxin A demyelination	AAV-mediated expression of CR2-Crry by RGCs reduced complement-dependent synaptic phagocytosis in the LGN and reduced visual dysfunction	(8)
Neuromyelitis optica	C5	Phase 3 Clinical Trial	Patients receiving eculizumab had a significantly lower risk of relapse compared to placebo	(151)
Stroke	C3	Intraluminal filament Middle Cerebral Artery Occlusion	B4-Crry reduced motor and cognitive deficits after stroke and reduced microglial phagocytosis of salvageable neurons	(85)
	C3	Microembolic Middle Cerebral Artery Occlusion, Intraluminal filament Middle Cerebral Artery occlusion	B4-Crry alone or in combination with tissue plasminogen activator prevented loss of hippocampal and perilesional synaptic density and reduced the risk of hemorrhagic transformation	(86)
Traumatic Brain Injury	C3	Controlled cortical impact	CR2-Crry, CR2-fH reduced chronic inflammation and neuronal loss and improved cognitive and functional recovery	(89)
Glaucoma	C3	DBA/2J mouse model of chronic glaucoma	AAV-mediated expression of CR2-Crry by RGCs reduced C3d deposition and RGC degeneration without affecting intraocular pressure	(10)
	C5	Experimental autoimmune glaucoma	Intravitreal injection of the anti-C5 monoclonal antibody BB5.1 reduced C3 levels and reduced apoptosis of RGCs, bipolar cells, and photoreceptor cells	(11)
Age-related Macular Degeneration	C5	Phase 2 Clinical Trial	Intravenous injection of eculizumab did not decrease the growth rate of geographic atrophy in dry AMD	(146)
	C3	Phase 2 Clinical Trial	Intravitreal administration of pegcetacoplan reduced the growth rate of geographic atrophy in dry AMD, but may carry increased risk of endophthalmitis and conversion to wet AMD	(12)
	Factor D	Phase 3 Clinical Trial	Intravitreal administration of anti-factor D blocking antibody lampalizumab did not reduce the growth rate of geographic atrophy in dry AMD relative to sham	(147)
	C5	Phase 2 Clinical Trial	Intravitreal injection of anti-C5 monoclonal antibody LFG316 did not decrease the growth rate of geographic atrophy in dry AMD	(152)
	C5 RNA	Phase 2a Clinical Trial	Intravitreal administration of the anti-C5 RNA aptamer ARC-1905 with a VEGF inhibitor improved visual acuity relative to patients only receiving the VEGF inhibitor	(152)
	C3bBb	Smoke exposure murine model	Intraperitoneal administration of CR2-fH preserved contrast threshold without affecting visual acuity and reversed retinal thinning and other morphologic changes relative to control	(144)
	C3bBb	Argon laser photocoagulation choroidal neovascularization	AAV-mediated expression of CR2-fH by RPE cells reduced complement activation and attenuates development of choroidal neovascularization	(153)
	C3bBb	Argon laser photocoagulation choroidal neovascularization	Intravitreal delivery of ARPE-19 cells stably expressing CR2-fH to the retina reduced complement activation and the development of choroidal neovascularization	(9)

*FH, factor H; AAV, adeno-associated virus; RGC, retinal ganglion cell; LGN, lateral geniculate nucleus; AMD, age-related macular degeneration; VEGF, vascular endothelial growth factor; RPE, retinal pigment epithelium*.

Complement-based therapeutics specific for different molecules or pathways would allow for effective treatment of disease while preserving the role of complement in other functions such as immune defense, retinal homeostasis, and other physiological functions of complement. Inhibition of complement at the C3 activation step knocks out all pathways and all enzymatically generated and assembled complement effector molecules. However, because of the multi-functionality of the complement system, a more targeted or selective approach at complement inhibition may be optimal, depending on how complement is involved in pathology. The C3 inhibitor pegcetacoplan, administered intravitreally, recently completed a phase 2 clinical trial in the treatment of geographic atrophy (GA) secondary to AMD with promising results (12). In this case, the localized administration reduced the chance of systemic complications, although endophthalmitis did occur in three participants receiving pegcetacoplan, and a significant portion of patients treated developed exudative AMD. Blockade of C5 has been a strategy for the treatment of other complement-mediated diseases in hematology (154) and nephrology (155) using an anti-C5 blocking antibody, eculizumab. Eculizumab is FDA approved for neuromyelitis optica, an autoimmune CNS disease with some clinical and pathological overlap with MS that is characterized by antibody-dependent complement activation (151). However, this and other C5-targeted strategies have failed in AMD. Anti-human C5 monoclonal antibodies [eculizumab (intravenous injection) (146) or LFG316 (intravitreal injections), summarized in (152)] did not decrease GA growth rate. Administration of an anti-C5 RNA aptamer [ARC 1905 (intravitreal injection)] with the vascular endothelial growth factor inhibitor ranibizumab preserved visual acuity better than ranibizumab alone in a phase 2a clinical trial [summarized in (152)]. Finally, lampalizumab, an anti-factor D alternative pathway inhibitor (156), failed to reduce the growth of GA in two phase 3 clinical trials for AMD when administered intravitreally (147), despite having achieved significance in a subgroup of dry AMD patients with complement factor I risk variants.

Another approach to limit systemic complement inhibition and limit potential off-target effects is to target complement inhibition specifically to the site of complement activation. Various protein inhibitors of complement fused to the C3 binding domain of complement receptor 2 (CR2) have been developed and tested in preclinical studies. The CR2 domain targets a complement inhibitor to sites of complement deposition, which prevents systemic effects of complement inhibition (157) and can function to block recognition of C3 opsonins by phagocytic cells. A CR2-factor H fusion protein, which is an alternative pathway-specific inhibitor, is effective when administered systemically in the smoke exposure dry AMD model (144). The same fusion protein, when expressed intraretinally after AAV-mediated (153) or cell-based delivery (9), reduced the growth of choroidal neovascularization in the laser-induced model of wet AMD. AAV-mediated intravitreal delivery of a CR2-Crry construct, which inhibits all complement pathways at C3 activation, provides neuroprotection to RGCs in murine models of glaucoma (10) and MS (8). Finally, intravitreal injection of a monoclonal antibody against mouse C5 also protected RGCs in a glaucoma model (11). These preclinical studies highlight the potential of targeted and/or localized delivery of complement inhibition with multiple modes of administration in reducing RGC death and visual loss in models of retinal and neurological disease.

## Conclusion

The complement system and phagocytosis play essential roles in sculpting neural circuits. However, in certain disease states the complement system is aberrantly activated and contributes to opsonization of functional and/or salvageable synapses, leading to irreversible damage. In this review, we focus on these processes in the afferent visual system, specifically the retina-LGN-primary visual cortex axis, although complement plays an active role in other regions of the brain in homeostasis and disease. The studies discussed here focus on the role of the complement system in the development of the visual system and in its degeneration in various disorders, including multiple sclerosis, glaucoma, and age-related macular degeneration. Although the studies discussed here primarily focus on the negative aspects of complement activation and opsonization, complement positively contributes to disease processes by tagging myelin and other debris for phagocytic removal, which in turn contributes to removal of damaged material and an anti-inflammatory microglial phenotype. We also included a review of complement inhibitory strategies, both systemic and targeted, that have been investigated in preclinical and clinical studies in the diseases discussed herein. A deeper understanding of the mechanistic role of complement and the contribution of specific pathways and activation products in different disease processes will assist in the development of smarter and more targeted approaches for the therapeutic application of complement inhibition in the clinic.

## Significance Statement

The visual system is one of the best understood neural circuits, with obvious functional importance. Over a decade ago, the centrality of the complement system in normal synaptic pruning in the afferent visual system was established. Around that time, the first links between polymorphisms in complement genes and risk of developing age-related macular degeneration were discovered. Since then, our understanding of the complement system's involvement in diseases of the brain and retina, and specifically its role in opsonizing synapses for removal by microglia, has expanded. Here, we review the current understanding of complement-mediated synaptic phagocytosis in the afferent visual system, specifically the retina and lateral geniculate nucleus, in normal development and disease. We focus on recent findings of synaptic phagocytosis in multiple sclerosis models, and review in general the involvement of complement in glaucoma and age-related macular degeneration. Lastly, we conclude with a brief look at complement inhibition in clinical trials and preclinical studies in diseases affecting the visual system.

## Author Contributions

DB and ST were responsible for conceptualization of the review topic. All authors contributed to the article and approved the submitted version.

## Conflict of Interest

ST is a cofounder and consultant for Q32Bio, a company developing complement inhibitors. ST and BR are inventors on patents that describe targeted complement inhibition. The remaining authors declare that the research was conducted in the absence of any commercial or financial relationships that could be construed as a potential conflict of interest.
